# Long-term effects of exercise interventions on physical activity in breast cancer patients: a systematic review and meta-analysis of randomized controlled trials

**DOI:** 10.1007/s00520-022-07485-6

**Published:** 2023-01-24

**Authors:** Siri Goldschmidt, Martina E. Schmidt, Karen Steindorf

**Affiliations:** 1 Division of Physical Activity, Prevention and Cancer (C110), German Cancer Research Center (DKFZ), Im Neuenheimer Feld 581, 69120 Heidelberg, Germany; 2 Medical Faculty of the University of Heidelberg, Im Neuenheimer Feld 672, 69120 Heidelberg, Germany

**Keywords:** Breast neoplasms, Maintenance, Cancer survivorship, Training

## Abstract

**Background:**

Benefits of exercise interventions for cancer patients are well established. This systematic review aimed to investigate the sustainability of exercise interventions with respect to physical activity behaviour of breast cancer patients in the longer term.

**Methods:**

The databases Pubmed, Cochrane, Embase, and Web of Science were systematically searched for randomized controlled trials (RCTs) investigating aerobic exercise, resistance exercise, or combined exercise interventions in breast cancer patients and assessing physical activity at least 2 months after the intervention. Random-effect models were used to calculate standardized mean differences (SMD).

**Results:**

A total of 27 RCTs with 4120 participants were included in the review, of which 11 RCTs with 1545 participants had appropriate data for the meta-analyses. Physical activity was mainly self-reported, and most exercise interventions were supervised. Exercise interventions tended to show a moderate significant effect up to 6 months for moderate to vigorous physical activity (SMD [95% CI] = 0.39 [0.07, 0.70]) and small, non-significant effects on total physical activity at 6 months (SMD [95% CI] = 0.14 [− 0.00, 0.28]) and up to 60 months after the intervention (SMD = 0.29 [-0.31, 0.90]). Differences between intervention characteristics, such as supervised versus unsupervised, were inconclusive due to the small number of RCTs.

**Conclusions:**

The physical activity behaviour in breast cancer patients remained improved for several months beyond the end of exercise interventions, but effects were small to moderate and diminished over time. Future studies should clarify how to maintain a healthy level of physical activity after completion of an exercise intervention.

**Supplementary Information:**

The online version contains supplementary material available at 10.1007/s00520-022-07485-6.

## Background

Physical activity (PA) is well known for reducing the risk of chronic diseases as well as side effects of cancer therapies and may improve prognosis and survival after cancer [1–6]. Thereby, PA recommendations for healthy adults and those for cancer patients and survivors are congruent, adapted to the respective individual limitations [5, 7]. Whilst PA comprises any movement of the body requiring energy, e.g. during everyday tasks or when walking the dog, the term exercise is used for planned, structured, and purposeful PA [8]. Exercise during and after chemo- and/or radio-therapy in breast cancer patients and survivors have been shown to be feasible and safe [3, 5, 9]. Further, exercise interventions were found to improve treatment-related side effects, quality of life and psychological health, physical fitness, and functioning [2, 3, 5, 10–16].

Despite these known benefits of PA and exercise, most breast cancer patients significantly reduce their PA during cancer treatment [11, 17–21]. Some months after completion of cancer treatment, improvements in PA behaviour were observed, but PA levels remained below those prior to diagnosis [17, 18, 20, 21].

Exercise interventions typically increase the PA behaviour over the duration of the intervention [11, 20, 22]. To maintain the positive effects achieved on physical and psychological outcomes, cancer survivors should continue to exercise after the end of the exercise intervention. However, so-far the sustainability of exercise interventions in terms of long-term PA behaviour is unclear, with widely varying results in the literature. A desirable sustainable intervention effect would be that cancer survivors continue the training after the end of the exercise intervention. However, a sustainable effect would also be if the exercise group has a significantly higher PA in the long term than the control group. Some previous qualitative reviews [14, 23] and a meta-analysis [24] considered intervention effects on longer term PA, but they included a broader range of interventions aiming to improve PA, i.e. not only exercise interventions but also behavioural or educative interventions without an exercise program. Further, a Cochrane review on interventions for promoting habitual exercise in cancer survivors concluded that long-term follow-up data are still too limited to answer the important question which interventions could promote PA for 12 months or longer [25].

Therefore, our aims were to perform a systematic review and quantitative analysis on the effect of exercise interventions on medium- and long-term PA behaviour in breast cancer patients, hereby considering also (1) different types of PA, (2) subjective or objective assessment of PA, and (3) different intervention characteristics (i.e. supervised vs. unsupervised training, training during or after cancer therapy, aerobic or resistance training).

## Methods

### Eligibility criteria

The review included only randomized controlled trials (RCT) with breast cancer patients. Study interventions were restricted to aerobic exercise, resistance training, or a combination of both. Moreover, the interventions had to have a duration of at least 4 weeks. They could be supervised or unsupervised. Interventions with only one or without any personal patient contact (e.g. providing only exercise prescriptions) were excluded, as were studies that focused on behaviour change only, and studies where the intensity of exercises were below three metabolic equivalents (METs), which is equivalent to light activity [26]. Further, eligible RCTs had to assess PA at baseline and at least at one follow-up time point more than 2 months post-intervention for both the intervention and the control groups. Subjective and objective assessments of PA were eligible. There were neither restrictions regarding the tumour or treatment stage of the participants nor the type of the control group.

### Literature search

**Fig. 1:**
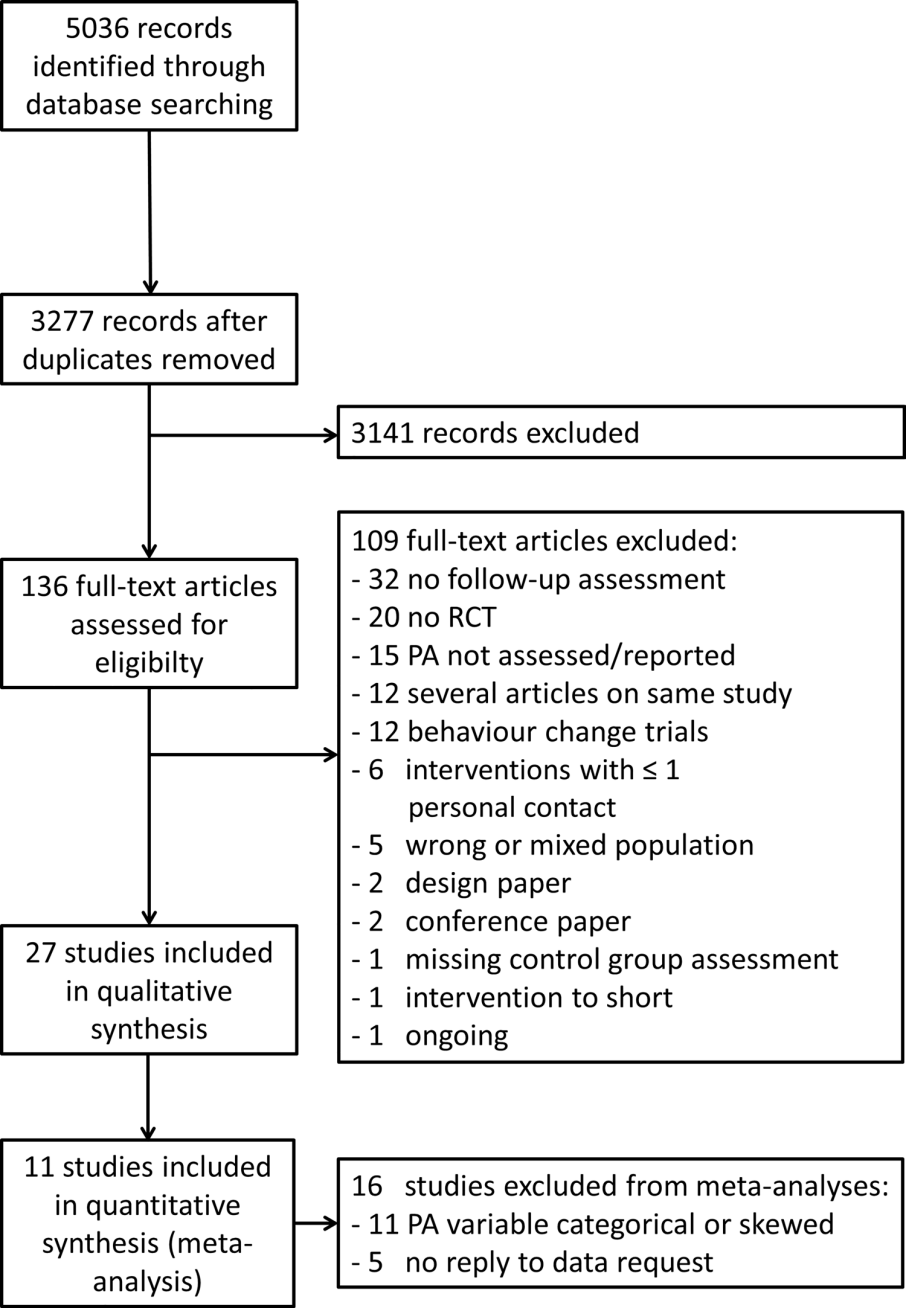
Flow chart

A systematic search was conducted in the databases Pubmed, Cochrane, Embase, and Web of Science until January 2022 following the Preferred Reporting of Systematic Reviews and Meta-analysis (PRISMA) guidelines. The search was limited to publications in English. Additionally, the reference lists of identified articles, reviews, and meta-analyses were checked. Every search result was screened on the title and, if tentatively relevant, on its abstract. If a study was found to be relevant, the full text was read by two reviewers. The flow chart of the included studies is presented in Fig. 1 and the search strategies are presented in supplement 1.

### Data extraction

Two reviewers independently extracted pre-intervention (baseline) and all follow-up data of each reported PA variable per group, including sample size (*N*), means (*M*), standard deviations (SD) or standard errors (SE), or median and inter-quartile range (IQR). In case of divergent extractions by the two reviewers, data were checked by a third reviewer to find a common solution. If information was missing or unclear, authors of the respective manuscripts were contacted.

Further, we extracted the follow-up time in months after the end of the intervention, the type and unit of the PA variable (e.g. total PA in MET*h/week, vigorous PA in min/week) and mode of assessment (e.g. subjective using a questionnaire or objective using accelerometers), the type of control group (e.g. usual care, waitlist control, stretching control), and characteristics of the study population such as mean age and stage of treatment. Regarding the intervention, several variables were of interest such as exercise type (i.e. aerobic exercise, resistance training, combination of both, walking intervention), setting (e.g. supervised, unsupervised home-based, or combination), frequency (scheduled number of training sessions per week), length of sessions, and duration of the intervention period. The intensity of each intervention was not specifically considered, but was of at least moderate intensity (see eligibility criteria).

### Risk of bias assessment

**Fig. 2:**
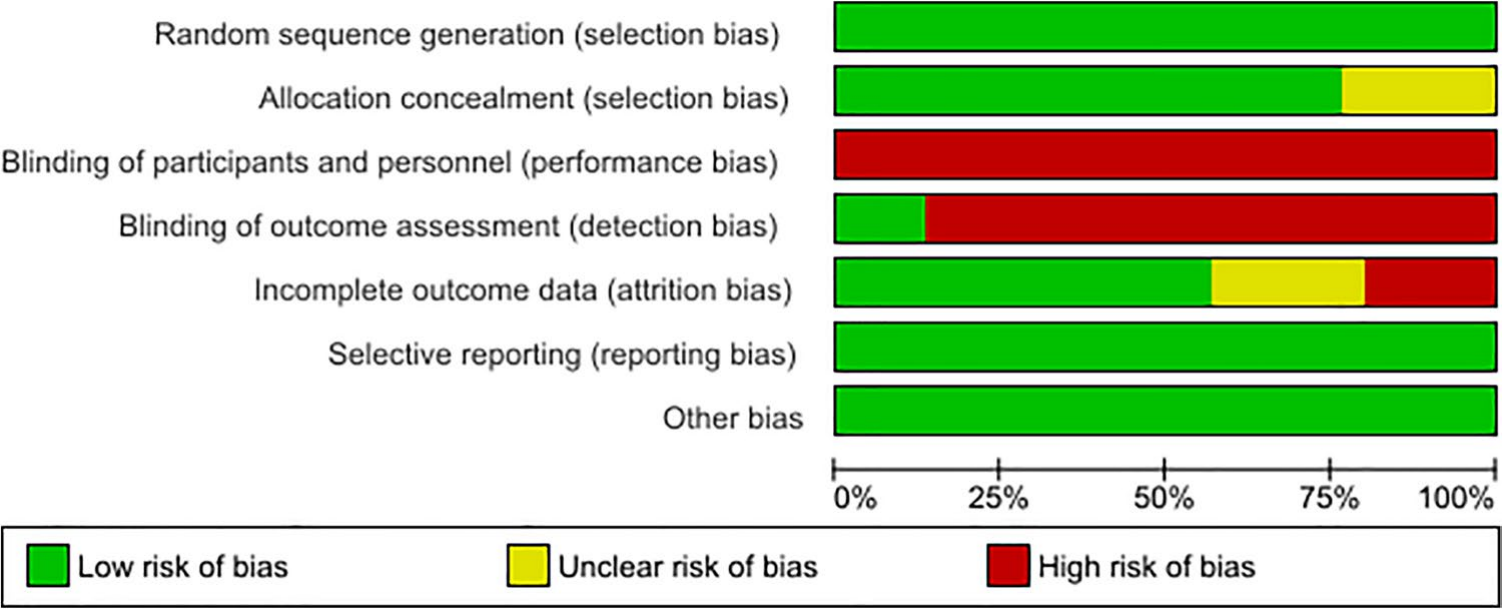
Risk of bias graph

**Fig. 3:**
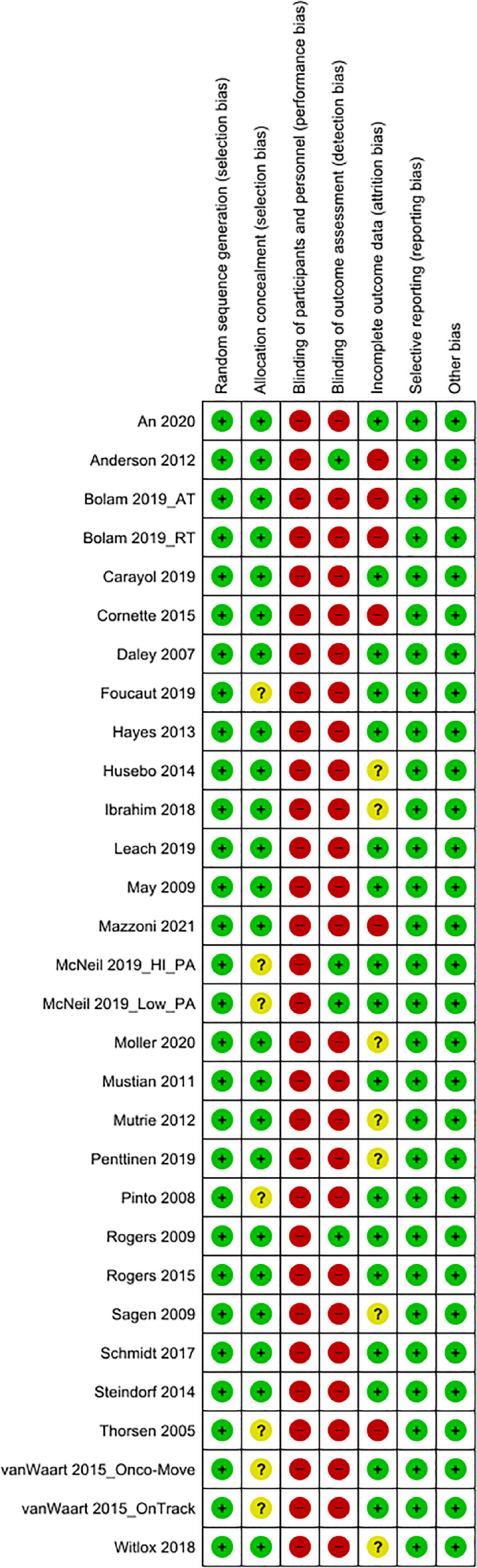
Risk of bias summary

The methodological quality of each study was examined according to the Cochrane risk of bias tool [27]. Two researchers independently performed the scoring. Divergent scoring was discussed and resolved together with a third reviewer. The results are summarized in Fig. 2 and described by study in Fig. 3.

Blinding of participants is not possible in exercise intervention studies. Thus, all RCTs included in this review were at high risk for performance bias. Likewise, in exercise intervention trials self-reported PA assessment is per-se unblinded; thus, these studies were judged at high risk of detection bias. In contrast, objectively assessed PA, e.g. by accelerometry, is considered low risk of detection bias.

To investigate the potential risk of publication bias, funnel plots were used for a visual assessment of whether small-study effects may be present in the meta-analyses. Egger’s test for funnel plot asymmetry is only recommended for meta-analyses that included at least 10 studies to allow a differentiation between chance and reality [28]. This was not possible for this meta-analysis, because no subgroup consisted of 10 studies. Thus, the heterogeneity between the studies was assessed with the Comprehensive Meta-Analysis Prediction intervals software (www.Meta-Analysis.com/Prediction). The prediction intervals represent the range of true effects for 95% of a comparable population [29]. Additionally, a sensitivity analysis was performed to investigate if studies with a higher risk of bias have an impact on the effect of exercise interventions on longer-term PA behaviour in breast cancer patients [30]. All studies consisting of three or more high risk of bias categories were excluded in this sensitivity analysis.

### Statistical analysis

Using SAS (version 9.4), the standardized mean differences (SMDs) with the respective 95% confidence interval (CI) were calculated for each study as the difference of the mean change from baseline to the respective follow-up measurement between the intervention and the control group divided by the pooled pre-test standard deviation [31]. Random effect models were computed using the Cochrane-Software RevMan 5.3.

Meta-analyses based on means are appropriate for data that are at least approximately normally distributed, and for data from very large trials. Yet, PA variables are often very skewed, especially considering MVPA, because often a high proportion of patients does not spend any or only little time with at least moderate intensity PA. Thus, in several studies, instead of mean (SD) of the PA variable rather median or percentage of participants meeting a certain activity level were given. Moreover, some publications reported means (SD) with the ratio mean/SD < 1.5. As this suggests a skewed distribution [28], these data were excluded from the meta-analysis. All study data that could not be quantitatively included in the meta-analyses were summarized and described qualitatively in the systematic review.

## Results

### Characteristics of the studies

Overall, 5036 articles were found in the four databases. After removing 1759 duplicates, the remaining 3277 articles were screened based on title and abstract, resulting in 136 articles that were considered suitable and read in their full length (Fig. 1). Of these, 27 articles comprising 4120 participants were deemed eligible and included in the systematic review. Table 1 summarizes the characteristics of the 27 included studies. They varied in the intervention duration (range: 4 to 52 weeks) and follow-up measurement time points after the completion of the intervention (range: 3 to 60 months).

**Table 1:** Characteristics of all included studies

Study	*N*, age			Intervention period	Intervention	Delivery mode	Duration (weeks)	Frequency, intensity, and further details	Follow-up (months)	Included in meta-analysis
An (2020) (Canada) [32]	STAN: *N* = 96 49.2 ± 8.4 HIGH: *N* = 101 50.1 ± 8.8 COMB: *N* = 104 50.5 ± 9.4			Adjuvant chemo-therapy	STAN: standard dose of aerobic exercise HIGH: a higher dose of aerobic exercise COMB: combined aerobic and resistance exercise	Supervised	12–18	STAN: 75 min/week of vigorous intensity/3 days/week for 25–30 min/session HIGH: 150 min/week of vigorous-intensity aerobic exercise/3 days/week for 50–60 min/session COMB: aerobic exercise of STAN group plus a standard resistance exercise program 3 days/week	24	No
Anderson (2012) (USA) [15]	<50: 50 to <65: 65 to < 75: >75:	IG 21 23 4 4	CG 23 19 7 8	Adjuvant chemo-/radio-therapy	IG: tailored exercise, lymphedema prevention, patient and diet education, and counselling CG: information materials	Supervised and not supervised home-based	24	IG: twice a week consisting of an aerobic warm up (5 min), 20-min full body workout using hand weights and resistance machines, 10-min stretching twice a week consisting of an aerobic warm up (5 min), 20-min full body workout using hand weights and resistance machines, 10-min stretching	15	No
Bolam (2019) (Sweden) [33]	RT: 58 53.4 ± 10.1 AT: 54 53.9 ± 9.2 CG: 48 54.1 ± 9.6			Adjuvant chemo-therapy	RT: combined resistance and aerobic training AT: aerobic training CG: usual care	Supervised	16	60 min/twice weekly RT: 8 machines, 2 sets, 8–12 repetitions at 70–80% of 1 repetition maximum (RM) + HIIT on a cycle ergometer: 3 × 3 min bouts at a rate of perceived exertion (RPE) of 16–18 with one-minute recovery between each bout AT: 20-min moderate intensity (RPE 13–15) and HIIT consisting of 3 × 3 min bouts at a RPE of 16–18 with one-minute recovery between each bout	20	Yes
Carayol (2019) (France) [34]	IG: 72 51.2 ± 10.9 CG: 71 52.1 ± 9.3			Adjuvant chemo-therapy	IG: 8–10 MET aerobic and resistance training/week CG: usual care	Supervised and not supervised home-based	26	IG: thrice weekly — one session muscle strengthening and two aerobic sessions (HR-related), increasing from 30–40 to 40–50 min per session	18	Yes
Cornette (2015) (France) [35]	IG: 22^*^ 52 (37–73) CG: 22 49 (37–68)			Adjuvant or neoadjuvant chemo-therapy	IG: aerobic and resistance training CG: usual care	Not supervised home-based	27	IG: 1 × /week individually tailored resistance training (2 × 8–12 reps) 2 × /week aerobic exercise according to HR at VT of CPET	6.75	Yes
Daley (2007) (USA) [36]	IG: 34 51.6 ± 8.8 Exercise placebo: 36 50.6 ± 8.7 CG: 38 51.1 ± 8.6			12–36 months after treatment completion	IG: aerobic exercise training Exercise placebo: light-intensity body conditioning (flexibility, stretching) CG: usual care	Supervised	8	IG: 3 × /week à 50-min moderate aerobic exercise at 65–85% of age-adjusted HR maximum and RPE of 12 to 13 + PA behaviour change	6	No
Foucaut (2019) (France) [37]	IG: 41^*^ 53.9 (26.2–71.5) CG: 19 49.4 (27.0–69.3)			Adjuvant chemo-therapy	IG: outdoor training and indoor fitness + dietary counselling CG: dietary and PA counselling according the guidelines for cancer survivors	Supervised If attendance was not possible, not supervised home-based	24	IG: twice weekly moderate-to-vigorous (≥ 3 MET) sessions of Nordic walking (60 min) and indoor fitness (45 min) consisting of aerobic-based exercises that involved the major muscles)	12	No
Hayes (2013) (Australia) [12]	IG: 207 51.7 ± 8.8 CG: 130 53.9 ± 8.3			After surgery	IG: combined aerobic- and resistance-based moderate activity CG: usual care	Supervised and not supervised	32	IG: supervised: one weekly session with an exercise physiologist Not supervised: 180 min + of aerobic- and resistance-based moderate activity per week to be accumulated on at least 4 days	12	No
Husebo (2014) (Norway) [38]	IG: 33 50.8 ± 9.7 CG: 34 53.6 ± 8.8			Adjuvant chemo-therapy	IG: combined aerobic and resistance training CG: usual care	Not supervised home-based	IG: 16.7 ± 7.6 CG: 17.6 ± 7.9	IG: 3 × /week resistance training + 30-min brisk walking daily	6	No
Ibrahim (2018) (Canada) [39]	IG: 29 CG: 30 Overall: 39.2 ± 5.0			Adjuvant radio-therapy	IG: combined strength, endurance, and stretching exercise for the upper body CG: usual care	Supervised and not supervised	12	IG: 6-week program of low-level cardiovascular and resistance exercises that progressed to a set of more advanced exercises for the remaining 6 weeks; strength: 8–12 repetitions, endurance: max 20 repetitions; at least once a week supervised and 2–3 times not supervised at home	18	No
Leach (2019) (USA) [40]	One-to-one: 12 51.9 ± 8.3 Group-based: 14 51.8 ± 9.2			Completed adjuvant treatment for breast cancer	One-to-one: combined aerobic and resistance training Group-based: combined aerobic and resistance training and PA behaviour change information/strategies	Supervised	8	One-to-one: 2 × /week à 40–55 min: 20–30-min aerobic exercise at 55–75% HRR, 20–25-min muscle strengthening and PA behaviour change information/strategies Group-based: 2 × /week à 40–55 min: 20–30-min aerobic exercise at 55–75% HRR, 20–25-min muscle strengthening	3	No
May 2009 (Netherlands) [41]	PT + CBT: 76 47.8 ± 10.5 PT: 71 49.9 ± 11.3			After completion of cancer treatment	PT + CBT: combined aerobic and resistance training with group sports and cognitive-behavioural therapy (CBT) PT: combined aerobic and resistance training with group sports	Supervised	12	PT + CBT: 2 × 2 sessions/week PT + once weekly CBT for 2 h: 30-min aerobic and 30-min strength training + 60-min group sports + cognitive-behavioural problem-solving per session PT: 2 × 2 sessions/week PT: 30-min aerobic and 30-min strength training + 60-min group sports per session	9	No
Mazzoni (2021) (Sweden) [42]	1. High intensity with BCT: 77 60 ± 12 2. Low intensity with BCT: 81 58 ± 12 3. High intensity without BCT: 71 57 ± 11 4. Low intensity without BCT: 72 60 ± 11			(Neo-)adjuvant treatment	Aerobic and resistance training with or without face-to-face self-regulatory behaviour change technique (BCT) sessions	Supervised and not supervised home-based	24	Supervised: resistance training — twice weekly - High intensity: alternated 3 × 6 and 3 × 10 repetition maximum - Low intensity: 3 × 12 repetitions at 50% of 6 RM and 3 × 20 repetitions at 50% of 10 RM Not supervised: aerobic training — twice weekly - High intensity: 20–40 min/session at 80–90% HRR twice per week - Low intensity: 150-min weekly continuous-based exercise at 40–50% HRR	12	No

The interventions were performed individually, except for one, which performed a group exercise programme [46]. Interventions included resistance training (6 studies), aerobic training (6 studies), compared aerobic with resistance training (3 studies), and a combination of aerobic and resistance training (12 studies). Of these, 7 interventions were unsupervised, all other interventions were at least partly supervised with or without additional home-based training.

Since only one study investigated walking as an intervention and outcome measure, walking could not be analysed separately from aerobic training.

### Meta-analysis

Of the 27 eligible studies, 16 studies had variables that were too skewed or did not provide means; thus, only 11 studies with 1545 participants, 850 in the intervention groups and 695 controls, were included in the quantitative meta-analyses (see Table 1).

In the studies included in the meta-analyses, PA was mainly assessed with questionnaires (see Table S2). Only four studies assessed PA objectively with accelerometry [33, 43, 49]. Reported PA variables were quite heterogenous including, for example, minutes per week spent with light, moderate, vigorous, or total PA; PA expressed in MET-hours per week; or dichotomous variables categorizing activity below or above a certain level. However, for calculating SMDs in the meta-analysis the unit of PA variables is irrelevant, and most PA variables could be classified either as “total PA” or as “MVPA” depending on the included activities. Moreover, follow-up time points after end of intervention covered a wide range. To compare the effects of the follow-up assessments, they were grouped according to their time in months after the intervention into approximately 3 months (including one study each with 2 and 4.5 months), 6 months (including one study with 6.2 months), 12–20 months, and 43.5–60 months (only 2 studies).

**Fig. 4:**
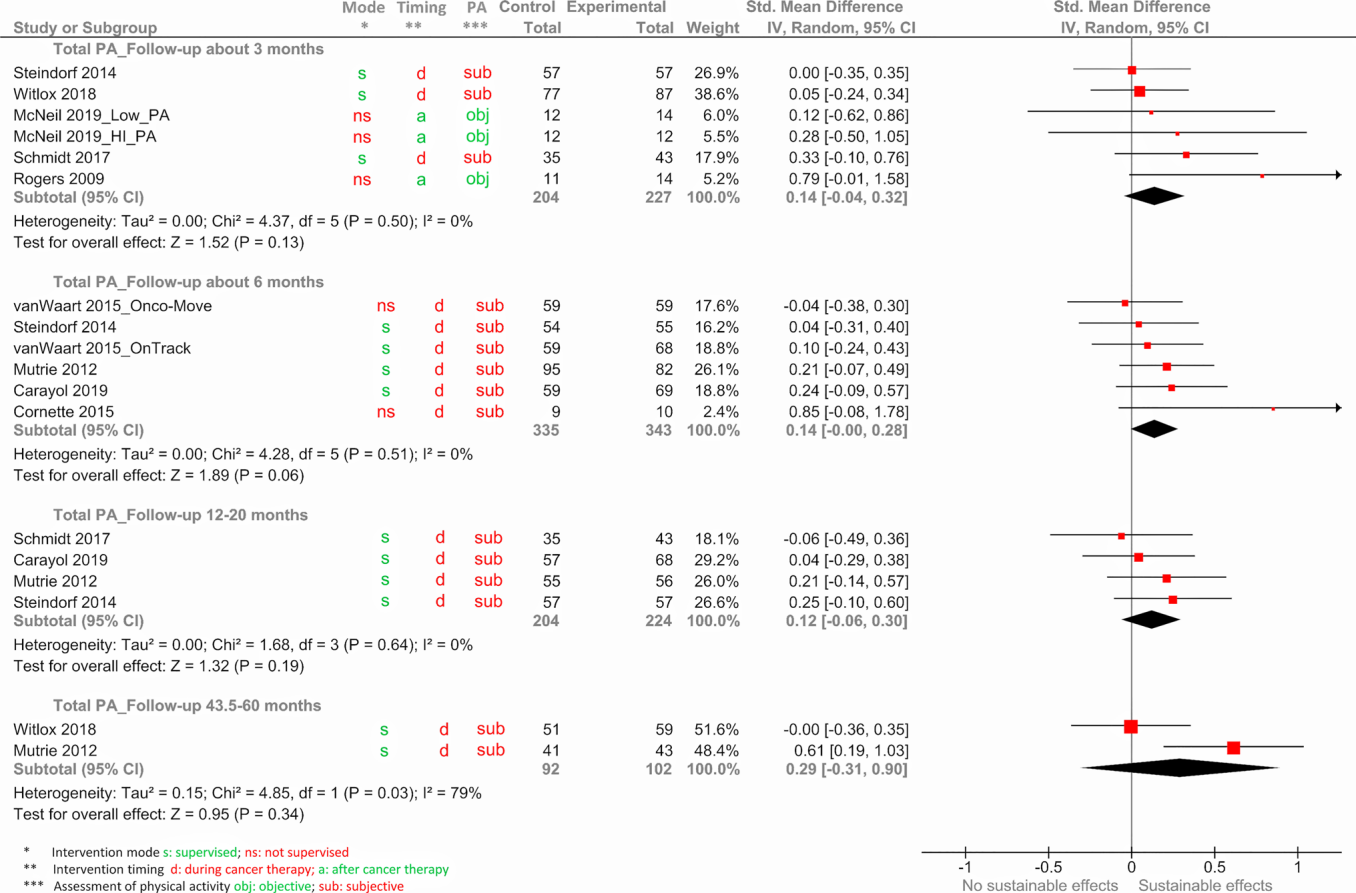
Forest plot for the sustainability of exercise interventions on the outcome total PA

Figure 4 shows the results of the meta-analyses of the outcome total PA for the different follow-up intervals.

There was a tendency to a sustainable effect of exercise interventions on the total PA behaviour up to 60 months after the end of the intervention (SMD [95% CI] = 0.29 [− 0.31, 0.90]; *p* = 0.34), but with only small effect sizes (SMDs between 0.12 and 0.29) and failing statistical significance (*p*-values between 0.06 and 0.34).

Sensitivity analyses, excluding the studies with more than three risk of bias categories judged as high, did not change the observations at around 3 months, 6 months, and 12 to 20 months after the intervention. No sensitivity analysis could be performed for the assessment 43.5 to 60 months post-intervention, because both concerning studies were at high risk of bias (see Fig. S1 in the supplement).

**Fig. 5:**
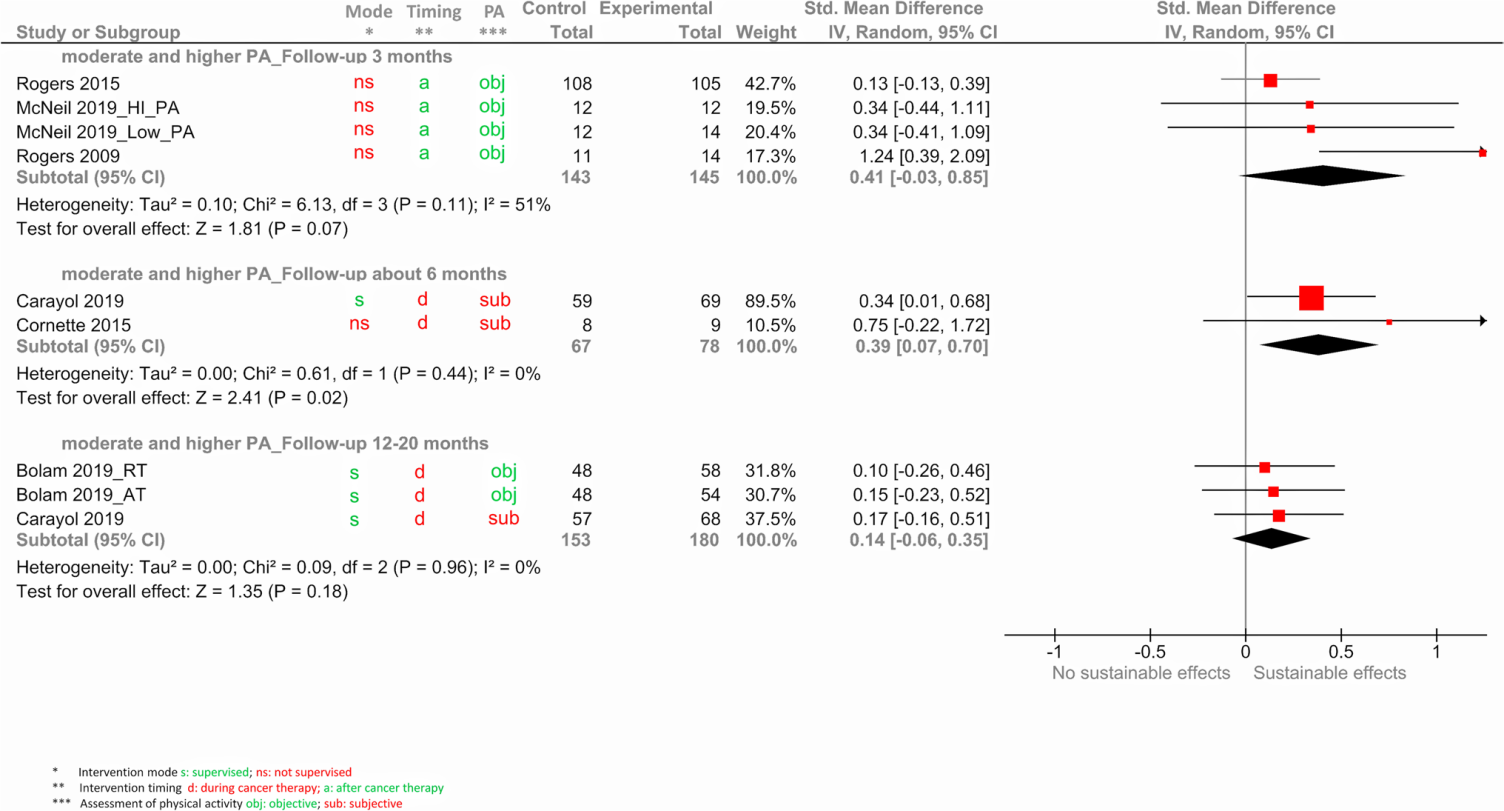
Forest plot for the sustainability of exercise interventions on the outcome MVPA

Figure 5 shows the results of the meta-analyses of the outcome MVPA for the different follow-up intervals.

There was a tendency for a small-to-moderate effect on MVPA 3 months post-intervention (0.41 [− 0.03, 0.85]; *p* = 0.07) and a similar effect after 6 months reaching significance (0.39 [0.07, 0.70]; *p* = 0.02) that decreased to a small effect 12 to 20 months after the intervention (0.14 [− 0.06, 0.35]; *p* = 0.18). These effects persisted in the sensitivity analyses excluding studies with higher level of bias (see Fig. S4 in the supplement).

The comparison of subjective and objective assessments of PA did not yield conclusive results. Total PA was assessed objectively in only 3 studies, with a follow-up of around 3 months, yielding a slightly higher effect with SMD of 0.38 [− 0.07, 0.82] compared to the remaining 3 studies at this time interval with subjective assessments (0.09 [− 0.10, 0.29]; supplement Fig. S2). Regarding MVPA, comparison of assessment mode was limited, because within each time interval all studies had used the same mode (supplement Fig. S5). Thus, moderate effects were seen for objectively assessed MVPA (0.41 [− 0.03, 0.85] around 3 months) as well as subjectively assessed MVPA (0.39 [0.07, 0.70] around 6 months). For the longer follow-up assessments similar effects were seen for subjective MVPA (0.17 [− 0.16, 0.51]) as well as objective MVPA (0.12 [− 0.14, 0.38]), but based on three studies only.

Stratification by supervised versus unsupervised interventions (supplement Figs. S3 and S6) showed partly somewhat higher effect sizes for unsupervised interventions, but due to the small number of studies and large confidence intervals the evidence is inconclusive. Comparisons of other intervention or population subgroups beyond those presented were not possible due to small subgroup sizes.

### Qualitative review of data not included in the meta-analyses

Study results regarding intervention effects on long-term PA that were not included into the meta-analyses due to missing information (*N*, mean, SD) [15, 45, 47, 51, 53], skewed PA variables [38, 39, 48], or only categorical PA results [12, 36, 37, 44] are described in Table 2. The table also includes additional relevant results, e.g. categorical data, of six studies [10, 20, 46, 50, 52, 54] that were included in the meta-analyses.

**Table 2:** Qualitative summary of important results of 12 studies that were not included in the meta-analyses and 6 studies included in the meta-analyses, but including additional important results

Study	Significant effect	Follow-up in months post-intervention	Results considering physical activity (PA) outcomes not included in the meta-analysis	
Anderson (2012) [15]	Yes	15	Participation in PA measured in pedometer steps was observed to be positively correlated with the distance covered in the 6-min walk test at the baseline assessment (*p* < 0.05). At 18 months, the IG covered significantly more meters in the 6-min walk test than the CG: adjusted mean (SE): 593.2 (13.0) vs. 558.9 (11.8), *p* = 0.0098	
Daley (2007) [36]	Yes	4	The proportion of participants who were inactive at baseline and increased their PA to become active at least 3 times per week at the end of the 8-week intervention period and 16 weeks later were significantly (*p* < 0.001) higher in the IG than in the usual care CG: 82% vs. 9% and 58% vs. 8%, respectively	
Foucaut (2019) [37]	No	12	Median duration (h/week) of MVPA (≥ 3 MET) and of VPA (≥ 4 MET) improved in both groups from BL to 12 months post-intervention with no significant group × time interaction (*p* = 0.40 and 0.11, respectively) MVPA median (min, max), h/week: IG: BL: 14.3 (2.7, 28.2), 12 months: 14.8 (3.1, 29.9) CG: BL: 14.3 (4.7, 27.3), 12 months: 16.2 (7.1, 55.5) VPA median (min, max), h/week: IG: BL: 0.4 (0.0, 8.3), 12 months: 1.7 (0.0, 10.8) CG: BL: 0.6 (0.0, 7.0), 12 months: 1.3 (0.2, 5.8)	
Hayes (2013) [12]	No	2	Median (Q1, Q3) MVPA minutes: Face to face: BL: 120 (5, 257.5), 2 months: 180 (0, 840) Telephone: BL: 7.5 (0, 127.5), 2 months: 120 (0, 1110) Usual care: BL: 45 (0, 125), 2 months: 120 (0, 1120)	
Husebo (2014) [38]	No	6	MET-min/week from IPAQ, mean (SD): IG: BL: 1333.66 (1367.67), 3 months: 2105.63 (2104.75) CG: BL: 1138.00 (1148.81), 3 months: 1844.94 (1555.35) There were no significant differences in changes in mean levels IG and CG The walking distance 6 months after chemo-therapy completion was significantly improved in both groups > > > > Data not included in the meta-analysis, because it seemed skewed (in part mean/SD < 1.5)	
Ibrahim (2018) [39]	?	15	The CG performed more PA than the IG 3 months after the intervention. Both groups returned to pre-diagnosis PA levels about 15 months after intervention > > > > Data not included in the meta-analysis, because it seemed skewed (in part mean/SD < 1.5)	
Moller (2020) [44]	Yes	9.3	Both groups (supervised exercise, pedometer intervention) significantly increased moderate PA during the intervention and maintained it until 9 months after the intervention. Thereby, the supervised exercise group had significantly higher PA values than the pedometer group	
Mustian (2009) [45]	Yes	3	Mixed population including 27 breast and 11 prostate cancer patients ANCOVA showed significantly more daily steps walked, minutes of resistance exercise, and resistance exercise days post-intervention and at the 3-month FU in IG than CG (all *p* values < 0.05) Daily steps, IG vs. CG: BL: 7222.2 ± 2691.3 vs. 5544.9 ± 2746.7 3-month FU: 12,878 ± 7570.1 vs. 5180.8 ± 3258.9 Daily resistance exercise (min), IG vs. CG BL: 1.16 ± 2.95 vs. 1.57 ± 4.73 3-month FU: 8.00 ± 10.26 vs. 0.73 ± 3.03 Days/week of resistance exercise, IG vs. CG BL: 0.21 ± 0.54 vs. 0.21 ± 0.63 3-month FU: 1.56 ± 2.50 vs. 0.12 ± 0.49	
Mutrie (2012) [46]	No	6, 18, 60	The significant increase in self-reported minutes of the intervention group regarding moderate PA per week during the intervention was not maintained until the 6-month follow-up 60 months after the intervention, the IG reported around 200-min PA per week more than the CG	
Penttinen (2019)^*^ [47]	No	48	No significant differences between IG and CG in change of PA from baseline to the 4-year follow-up. In contrary, CG patients were actually more physically active than IG patients after the end of the 1-year intervention	
Pinto (2008) [48]	Yes	3, 6	Significant between-group differences in MVPA at the end of a 12-week intervention vanished 3 months post-intervention, but were significant again 6 months post-intervention > > > > Data not included in the meta-analysis, because it seemed skewed (in part mean/SD < 1.5)	
Rogers (2015) [50]	Yes	3	At the 3-month follow-up, participants of the IG were significantly more likely to meet PA recommendations than CG (accelerometry assessed PA: OR = 2.4 (95% CI: 1.1–5.3), self-reported PA: OR = 4.8 (95% CI: 2.3–10.0)) Proportions meeting PA recommendations:	
			Accelerometry	IG: BL: 49.8%, 3 months: 67.4% CG: BL: 49.8%, 3 months: 53.6%
			Self-report	IG: BL: 8.7%, 3 months: 45.6% CG: BL: 2.8%, 3 months: 17.7%
Sagen (2009) [51]	No	24	No group differences regarding PA at the 2-year follow-up measurement	
Schmidt (2015) [55] and Steindorf (2014) [52]^**^	No	12	Proportions of patients self-reporting any exercise at 12 months post-intervention were similar to pre-diagnosis levels in IG and CG: IG: pre-diagnosis: 67.5%, 12 months: 68.0% CG: pre-diagnosis: 67.0%, 12 months: 72.0% However, the resistance training intervention appeared to influence the type of exercise performed, with strength exercise being the most common type of exercise at follow-up in the resistance exercise IG, conducted more frequently than in the CG	
Thorsen (2007) [53]	No	12	At the 6- and 12-month follow-ups, no intergroup differences in types of performed activities or the numbers of activities per patients were observed	
Van Waart (2015) [10]	No	6	No significant group differences observed between OnTrack, OncoMove, and UC neither post-intervention nor at the 6-month FU regarding PA	
Witlox (2018) [54]	Yes	4.5, 43.5	The number of participants meeting the aerobic exercise guidelines was similar in IG and CG 4.5 months post-intervention, but 43.5 months post-intervention more patients achieved aerobic exercise guidelines in IG than CG: BL: 54.4% vs. 51.7% 4.5-month FU: 30.0% vs. 33.0% 43.5-month FU: 72.1% vs. 64.3% 43.5 months post-intervention: IG reported significantly more MVPA than CG (between-group difference 141.46 min/week, 95% CI: (1.31, 281.61), effect size = 0.22) [population including besides breast cancer also few colon cancer patients]	

*BL* – baseline, *IG* – intervention group, *CG* – control group, *FU* – follow-up, *MET* – metabolic equivalent of task, *MVPA* – moderate-to-vigorous physical activity, *SD* – standard deviation, *PA* – physical activity, *Q1* – first quartile, *SE* – standard error, *UC* – usual care, *VPA* – vigorous PA.

^*^Also published in Vehmanen (2021) [56]

^**^Published in Schmidt (2017) [20]

Commonly, the PA behaviour remained unchanged or improved from the baseline measurement during and beyond the end of the exercise intervention. In some studies, however, PA also improved in the control group, resulting in similar PA changes over and beyond the intervention period and, thus, in non-significant group differences [10, 12, 37, 38, 44, 47]. Some studies reported improvements in their intervention groups that exceeded those of the control group, but were not maintained in the longer term after the end of the intervention [20, 39, 51–53]. Of these, three studies reported a return to the pre-diagnosis levels [20, 39, 52].

The remaining seven studies reported a continuous superior PA behaviour in the intervention group (IG) compared to the control group (CG) post-intervention and in the longer term [15, 36, 45, 50] or a superior performance in the IG post-intervention that vanished in the first follow-up, but appeared again some months [48] or years after the intervention [46, 54].

Table 3 summarizes all studies that compared different exercise interventions with each other. The comparison of aerobic exercise in two different intensities with a combined aerobic and resistance exercise (COMB) group showed no statistically significant differences between both aerobic groups in meeting the aerobic and resistance exercise guidelines in the follow-up periods, but both groups were superior in meeting the aerobic exercise guidelines compared to the COMB group [32]. The COMB group was superior in meeting the resistance exercise guidelines [32]. Studies comparing exercise interventions with an additional cognitive component observed conflicting results [41, 42]. May and colleagues [41] did not observe additional PA improvements, whereas Mazzoni and colleagues [42] observed more sustainable PA levels in patients that receive PA with an additional self-regulatory behaviour change technique.

**Table 3:** Qualitative summary of results of studies comparing different exercise interventions

Study	Follow-up in months post-intervention	Results considering PA outcomes after different interventions				
An (2020) [32]	6, 12, 24	Meeting resistance exercise guidelines: COMB significantly superior to HIGH at 6 and 24 months				
			STAN vs. HIGH vs. COMB			
		BL: 6 months: 12 months: 24 months:	21.9% vs. 18.8% vs. 23.1% 42.4% vs. 32.6% vs. 52.0% 39.6% vs. 36.8% vs. 45.9% 39.3% vs. 28.4% vs. 42.3%			
		Meeting aerobic exercise guidelines: HIGH significantly superior to COMB at 6 months No significant difference between STAN and HIGH				
			STAN vs. HIGH vs. COMB			
		BL: 6 months: 12 months: 24 months:	31.3% vs. 28.7% vs. 30.8% 62.0% vs. 64.2% vs. 49.5% 67.0% vs. 63.2% vs. 67.3% 60.7% vs. 56.8% vs. 54.6%			
Hayes (2013) [12]	2	Median (Q1, Q3) total PA minutes:				
			Face to face vs. telephone vs. usual care			
		BL:	120 (5, 257.5) vs. 7.5 (0, 127.5) vs. 45 (0, 125)			
		2 months:	180 (0, 840) vs. 120 (0, 1110) vs. 120 (0, 1120)			
Leach (2019) [40]	3	Patients performing MVPA (≥ 5 days of any combination of walking, moderate- or vigorous-intensity activities, and total PA ≥ 600 MET-min/week):				
			Individually/one-to-one vs. group dynamic-based exercise			
		BL:	42% vs. 50%			
		3 months:	54.5% vs. 91.7%			
		Total PA (MET-min/week), mean (SD)				
			Individually/one-to-one vs. group dynamic-based exercise			
		BL:	1655.8 (1663.2) vs. 1699.5 (1785.9)			
		3 months:	2072.1 (1918.8) vs. 4991.6 (5812.80)			
		> > > > Data not included in the meta-analysis, because it seemed skewed (in part mean/SD < 1.5)				
May (2009) [41]	6, 9	No significant group differences in PA were observed between PT and PT + CBT. Compared to baseline, PA was significantly improved in PT and PT + CBT post-intervention and in the 6-month follow-up, and the post-intervention PA values for both groups were maintained until 9-month post-intervention Thus, adding CBT to a supervised group-based self-management PT did not further enhance the beneficial effects of physical training alone				
Mazzoni (2021) [42]	12	Participants, *N* (%), maintaining PA levels at 12-month follow-up in relation to post-intervention:				
			With BCT		Without BCT	
			HI	LMI	HI	LMI
		Aerobic only	61 (79)	54 (67)	40 (56)	51 (71)
		Moderate	35 (45)	30 (37)	28 (39)	27 (38)
		Vigorous	47 (61)	46 (57)	26 (37)	42 (58)
		Moderate-to-vigorous	35 (45)	29 (36)	26 (37)	27 (38)
		Resistance only	2 (3)	1 (1)	2 (3)	0 (0)
		Aerobic and resistance	3 (4)	4 (5)	4 (6)	1 (1)
		More participants with self-regulatory behaviour change techniques (BCTs) maintained their PA than those without BCT (1.8 times the odds)				
vanWaart (2015) [10]	6	No significant group differences between OnTrack, OncoMove, and UC neither post-intervention nor at the six-month FU regarding PA were observed				

*BCT* – behaviour change techniques, *BL* – baseline, *CBT* – cognitive-behavioural therapy, *CG* – control group, *COMB* – high dose of combined aerobic and resistance exercise, *HI* – high intensity, *HIGH* – high dose of aerobic exercise, *IG* – intervention group, *FU* – follow-up, *LMI* – low-to-moderate intensity, *MET* – metabolic equivalent of task, *MVPA* – moderate-to-vigorous physical activity, *N* – number, *SD* – standard deviation, *PA* – physical activity, *PT* – physical training, *Q1* – first quartile, *SD* – standard deviation, *STAN* – standard dose of aerobic exercise, *UC* – usual care.

Comparing supervised interventions with unsupervised interventions showed also conflicting results. One study observed that individual supervised interventions appeared to be slightly superior to other supervised and unsupervised interventions [12] and one study did not observe any group differences [10].

## Discussion

The aim of this systematic review and meta-analysis was to investigate the impact of exercise interventions on the PA behaviour of breast cancer patients in the longer term. Hereby, we considered (1) different types of PA (i.e. total PA, MVPA), (2) the mode of PA assessment (i.e. subjective or objective), and (3) different intervention characteristics (i.e. supervised/unsupervised training). The quantitative as well as qualitative analysis showed that the effects of exercise interventions on PA can persist beyond termination of the interventions.

However, the effects on total PA revealed by the meta-analyses were small throughout all follow-up intervals up to 60 months post-intervention and failed to reach statistical significance. One reason might be that total PA included also physical activities beyond the exercise targeted by the considered interventions (e.g. occupational activity). Effects on MVPA up to 6 months post-intervention were somewhat larger, partly reaching statistical significance, but decreased to a small effect again at 12 to 20 months post-intervention. The studies that were not included in the meta-analyses and only qualitatively analysed showed also some sustainable effects of the exercise interventions on the amount of PA behaviour in the longer term.Our findings are in line with a previous meta-analysis which concluded that interventions can increase MVPA behaviour of cancer survivors at least 3 months after completing the intervention [24]. However, that meta-analysis included not only exercise interventions (i.e. where patients are asked to conduct aerobic and/or resistance exercise) but also interventions that aimed to improve PA by behaviour change techniques such as providing educational material, counselling by phone calls, or providing a pedometer. Therefore, besides updating the previous review by more recent publications, we refined the analyses by focussing on exercise interventions, more defined follow-up time points, and considering different types of PA. Two qualitative systematic reviews on interventions aiming to increase PA amongst breast cancer patients could not draw clear conclusions on long-term PA behaviour due to limited number and heterogeneity of the trials [14, 23].

The observation of only small to moderate effects of an exercise intervention on longer-term PA may be in part attributed to the PA behaviour of the control groups that sometimes also increased during or after the intervention period [10, 12, 37, 38, 47]. One possible explanation may be the selection bias that is inherent in intervention studies, namely that mostly those patients who have already been interested in PA are more prone to participate [37]. Further, some studies were waitlist-control trials, offering the exercise intervention after the end of the trial also to the control participants. Also, the study-related repeated PA assessments by questionnaires or fitness trackers as well as physical fitness testing might trigger an increase in PA amongst patients randomized to the control group.

There is no gold standard for assessing PA and manifold methods were used across the studies. Sometimes it is argued that objective assessment, e.g. by accelerometry, is more precise and may avoid overreporting of PA behaviour that may be associated with self-report assessment by questionnaires. However, objective assessments have also limitations, e.g. do not always precisely record activities such as bicycling or swimming as was mentioned by Rogers and colleagues [49]. In our meta-analyses there was no clear difference between effects on objectively assessed PA and effects on subjectively assessed PA. This can be drawn back to the low number of available studies assessing PA objectively, and therefore, this conclusion needs to be interpreted with caution.

Supervised exercise interventions appear to exert larger effects than unsupervised exercise interventions regarding patient-reported outcomes such as fatigue, anxiety, depressive symptoms, and health-related quality of life [3]. In contrast, in terms of sustainable effects on PA our analyses did not reveal a clear advantage of either intervention type, i.e. supervised or unsupervised. This may be related to the low quantity of available studies, of which several entailed a combined supervised and unsupervised exercise intervention. Whilst supervision seems to be important for training adherence and might result in a higher dose of exercise possibly due to more attention, motivation, and reinforcement [25], these advantages of supervision fade after termination of the intervention, potentially leaving the patients lost in the transition to practicing PA and exercise on their own [20, 48].

The majority of the identified studies conducted the exercise intervention during cancer therapy, thus limiting comparisons by timing of the intervention. The timing might play a role in the maintenance of PA post-intervention. A cancer therapy phase is a special circumstance, in which many patients are on sick leave and focus more on healthy behaviour. This may promote the uptake of exercise training. Yet, not only the uptake, but also the maintenance of PA in cancer survivors is a crucial concern. After completion of the intervention and the therapy, however, when cancer survivors return to their former social/familial and occupational everyday life, they often seem to also return to their pre-diagnosis physical activity (respectively, inactivity) behaviour [20, 57]. Thus, both seem important, i.e. fostering physical exercise during cancer therapy and additionally offering exercise programs for cancer survivors post-therapy.

Due to a low number of studies per subgroup our comparisons of intervention characteristics and PA assessment method were limited, and thus, no clear advantages of either compared approaches showed a clear advantage. Therefore, the presented tendencies of effects need to be interpreted with caution. Furthermore, it was not possible to analyse group-based vs. individual training, aerobic vs. resistance vs. combined training, to compare different intensities of exercise, or differentiate by patients or treatment characteristics. However, some studies suggest that the type of exercise in the intervention has a sustainable impact on the type of exercising in the longer term. An and colleagues observed a significant superiority of the group with higher-intensity aerobic exercise regarding the percentage of participants meeting the aerobic exercise guidelines compared to the combined aerobic and resistance exercise group [32]. On the other hand, significantly more participants of the combined aerobic and resistance exercise group met the resistance exercise guidelines at the 6- and 24-month follow-up. Similarly, Schmidt and colleagues [20] found that 12 months after a resistance exercise intervention participants engaged more in resistance exercise compared to the year prior to the diagnosis, whereas there was no such increase in the relaxation control group. Some data on the impact of the exercise intensity is provided by An et al., who found no significant differences in follow-up PA between a standard dose of aerobic exercise, which was described as 25 to 30 min of aerobic exercise, and a high-intensity aeobic exercise intervention with twice the standard dose [32]. A review of Kampshoff et al. identified 6 studies that focused on determinants of exercise maintenance after completion of an intervention, which yielded no clear association with demographic and clinical factors [57].

Our finding that most exercise interventions have only limited sustainable effects on PA behaviour suggests that additional approaches may be necessary to increase PA in the long term. May and colleagues added cognitive-behavioural therapy to a physical training and compared it with the physical training alone [41]. The RCT showed no significant group difference regarding PA maintenance. However, in both groups PA increased during the intervention and PA levels were maintained up to 12 months post-intervention [41]. A possible reason might have been that the physical training was offered in a group format providing opportunities for social interaction and group support that might improve self-efficacy. Yet, a review examining the role of group dynamics in exercise and PA interventions concluded that its additional benefits for increasing PA in cancer survivors are still unclear and that it needs to be further investigated how to optimally use the potential of group dynamic strategies [40]. A recently published follow-up of the Phys-Can study including 301 survivors of breast, colorectal, or prostate cancer found a significant effect of adding behaviour change techniques (BCT) to exercise interventions in terms of improved PA maintenance at 12 months post-intervention [42]. These self-regulatory BCTs comprised goal setting, review of behavioural goals, self-monitoring, action planning, and problem solving and were provided face-to-face supervision in the resistance training sessions on a maximum of 9 occasions as well as at follow-up prompts by study coaches at 3 and 9 months after the exercise intervention. A meta-analysis suggested that PA-promoting interventions relying on BCTs congruent with (social) learning theory, such as using prompts and rewards and setting graded tasks, might be successful in promoting PA in cancer survivors [58]. Similarly, a review and meta-analysis considering interventions to promote PA in healthy inactive adults found that maintenance of PA was associated with using action planning, instruction on how to perform the behaviour, prompts and cues, behaviour practice and rehearsal, graded tasks, and self-reward [59]. Moreover, a recent RCT investigating different approaches to promote PA in 161 breast cancer survivors found that phone calls from peer mentors and text messaging improved PA maintenance [60]. Thus overall, integrating some social, cognitive, and behavioural components in exercise interventions may be important to maintain the recommended PA levels over the long term and should be further investigated in future studies. Hereby, eHealth and wearables might also be a beneficial approach.

### Strengths and limitations

Limitations of the meta-analyses include the small number of studies with appropriate data that did not allow further exploration of the potential impact of setting, type, intensity, and frequency of exercise. Moreover, for most included RCTs the reporting of longer-term participation in resistance exercises was scarce or lacking, representing a major gap in current literature. Likewise, control group contamination, i.e. control group participants becoming active, was insufficiently reported.Further, PA assessment was very heterogenous and generally has its limitations irrespective of methods used.

## Conclusion

Exercise interventions were found to be sustainable in terms of improved PA behaviour for several months beyond the end of the intervention by increasing especially activities of moderate to vigorous intensity. However, the effects were only of small to moderate size and appeared to decrease over time. Future studies should clarify how sustainability could be achieved. There are indications that integrating social, cognitive, and behavioural components in exercise interventions may contribute to long-term PA maintenance in cancer survivors.

## Supplementary Information

Below is the link to the electronic supplementary material.

Supplementary file1 (TIF 104275 KB)

Supplementary file2 (TIF 106103 KB)

Supplementary file3 (TIF 96118 KB)

Supplementary file4 (TIF 109711 KB)

Supplementary file5 (TIF 102930 KB)

Supplementary file6 (TIF 103325 KB)

Supplementary file7 (DOCX 21 KB)

Supplementary file8 (DOCX 43 KB)

## Data Availability

No new data were created or analysed in this study. Data sharing is not applicable to this article.

## References

[1] Global Recommendations on Physical Activity for Health. Geneva: World Health Organization 2010 26180873

[2] Lahart IM, Metsios GS, Nevill AM, Carmichael AR (2018) Physical activity for women with breast cancer after adjuvant therapy. Cochrane Database Syst Rev 1(1):CD011292 10.1002/14651858.CD011292.pub2 PMC6491330 29376559

[3] Campbell KL, Winters-Stone KM, Wiskemann J, May AM, Schwartz AL, Courneya KS (2019). Exercise guidelines for cancer survivors: consensus statement from International Multidisciplinary Roundtable. Med Sci Sports Exerc.

[4] Patel AV, Friedenreich CM, Moore SC, Hayes SC, Silver JK, Campbell KL (2019). American College of Sports Medicine Roundtable Report on physical activity, sedentary behavior, and cancer prevention and control. Med Sci Sports Exerc.

[5] Schmitz KH, Courneya KS, Matthews C, Demark-Wahnefried W, Galvão DA, Pinto BM (2010). American College of Sports Medicine roundtable on exercise guidelines for cancer survivors. Med Sci Sports Exerc.

[6] McTiernan A, Friedenreich CM, Katzmarzyk PT, Powell KE, Macko R, Buchner D (2019). Physical activity in cancer prevention and survival: a systematic review. Med Sci Sports Exerc.

[7] WHO (2020) WHO guidelines on physical activity and sedentary behaviour. Geneva: World Health Organization. Licence: CC BY-NC-SA 3.0 IGO.2020

[8] Caspersen CJ, Powell KE, Christenson GM (1985). Physical activity, exercise, and physical fitness: definitions and distinctions for health-related research. Public Health Rep.

[9] Wolin KY, Schwartz AL, Matthews CE, Courneya KS, Schmitz KH (2012). Implementing the exercise guidelines for cancer survivors. J Support Oncol.

[10] van Waart H, Stuiver MM, van Harten WH, Geleijn E, Kieffer JM, Buffart LM (2015). Effect of low-intensity physical activity and moderate- to high-intensity physical exercise during adjuvant chemotherapy on physical fitness, fatigue, and chemotherapy completion rates: results of the PACES randomized clinical trial. J Clin Oncol.

[11] An K-Y, Kang D-W, Morielli AR, Friedenreich CM, Reid RD, McKenzie DC (2020). Patterns and predictors of exercise behavior during 24 months of follow-up after a supervised exercise program during breast cancer chemotherapy. Int J Behav Nutr Phys Act.

[12] Hayes SC, Rye S, Disipio T, Yates P, Bashford J, Pyke C (2013). Exercise for health: a randomized, controlled trial evaluating the impact of a pragmatic, translational exercise intervention on the quality of life, function and treatment-related side effects following breast cancer. Breast Cancer Res Treat.

[13] Gokal K, Wallis D, Ahmed S, Boiangiu I, Kancherla K, Munir F (2016). Effects of a self-managed home-based walking intervention on psychosocial health outcomes for breast cancer patients receiving chemotherapy: a randomised controlled trial. Support Care Cancer.

[14] Abdin S, Lavallée JF, Faulkner J, Husted M (2019). A systematic review of the effectiveness of physical activity interventions in adults with breast cancer by physical activity type and mode of participation. Psychooncology.

[15] Anderson RT, Kimmick GG, McCoy TP, Hopkins J, Levine E, Miller G (2012). A randomized trial of exercise on well-being and function following breast cancer surgery: the RESTORE trial. J Cancer Surviv.

[16] Courneya KS, McKenzie DC, Mackey JR, Gelmon K, Friedenreich CM, Yasui Y (2013). Effects of exercise dose and type during breast cancer chemotherapy: multicenter randomized trial. JNCI: J Natl Cancer Inst.

[17] Devoogdt N, Van Kampen M, Geraerts I, Coremans T, Fieuws S, Lefevre J (2010). Physical activity levels after treatment for breast cancer: one-year follow-up. Breast Cancer Res Treat.

[18] Littman AJ, Tang M-T, Rossing MA (2010). Longitudinal study of recreational physical activity in breast cancer survivors. J Cancer Surviv.

[19] Bock C, Schmidt ME, Vrieling A, Chang-Claude J, Steindorf K (2013). Walking, bicycling, and sports in postmenopausal breast cancer survivors—results from a German patient cohort study. Psychooncology.

[20] Schmidt M, Wiskemann J, Ulrich C, Schneeweiss A, Steindorf K (2017). Self-reported physical activity behavior of breast cancer survivors during and after adjuvant therapy: 12 months follow-up of two randomized exercise intervention trials. Acta Oncol.

[21] De Groef A, Geraerts I, Demeyer H, Van der Gucht E, Dams L, de Kinkelder C (2018). Physical activity levels after treatment for breast cancer: two-year follow-up. Breast.

[22] Bluethmann SM, Vernon SW, Gabriel KP, Murphy CC, Bartholomew LK (2015). Taking the next step: a systematic review and meta-analysis of physical activity and behavior change interventions in recent post-treatment breast cancer survivors. Breast Cancer Res Treat.

[23] Spark LC, Reeves MM, Fjeldsoe BS, Eakin EG (2013). Physical activity and/or dietary interventions in breast cancer survivors: a systematic review of the maintenance of outcomes. J Cancer Surviv.

[24] Grimmett C, Corbett T, Brunet J, Shepherd J, Pinto BM, May CR (2019). Systematic review and meta-analysis of maintenance of physical activity behaviour change in cancer survivors. Int J Behav Nutr Phys Act.

[25] Turner RR, Steed L, Quirk H, Greasley RU, Saxton JM, Taylor SJ et al (2018) Interventions for promoting habitual exercise in people living with and beyond cancer. Cochrane Database Syst Rev 9(9):Cd010192 10.1002/14651858.CD010192.pub3 PMC6513653 30229557

[26] Ainsworth BE, Haskell WL, Herrmann SD, Meckes N, Bassett DR, Tudor-Locke C (2011). 2011 Compendium of Physical Activities: a second update of codes and MET values. Med Sci Sports Exerc.

[27] Higgins JPT SJ, Page MJ, Elbers RG, Sterne JAC (2021) Chapter 8: Assessing risk of bias in a randomized trial. In: Higgins JPT, Thomas J, Chandler J, Cumpston M, Li T, Page MJ, Welch VA (eds) Cochrane handbook for systematic reviews of interventions version 6.2 (updated February 2021). Cochrane. Available from https://training.cochrane.org/handbook/current/chapter-08. Accessed Dec 2021

[28] Higgins JPT GS (2011) Cochrane Handbook for Systematic Reviews of Interventions 2011. The Cochrane Collaboration. Version 5.1.0 [updated March 2011]

[29] Borenstein M, Higgins JP, Hedges LV, Rothstein HR (2017) Basics of meta-analysis: I2 is not an absolute measure of heterogeneity. Res Synth Methods 8(1):5–18 10.1002/jrsm.1230 28058794

[30] Schmucker C, Nothacker M, Möhler R, Meerpohl J (2017) Bewertung des Verzerrungsrisikos von systematischen Übersichtsarbeiten: ein Manual für die Leitlinienerstellung. http://www.cochrane.de/de/review-bewertung-manual. Accessed Dec 2021

[31] Morris S (2008). Estimating effect sizes from pretest-posttest-control group designs. Organ Res Methods.

[32] An KY, Morielli AR, Kang DW, Friedenreich CM, McKenzie DC, Gelmon K (2020). Effects of exercise dose and type during breast cancer chemotherapy on longer-term patient-reported outcomes and health-related fitness: a randomized controlled trial. Int J Cancer.

[33] Bolam KA, Mijwel S, Rundqvist H, Wengström Y (2019). Two-year follow-up of the OptiTrain randomised controlled exercise trial. Breast Cancer Res Treat.

[34] Carayol M, Ninot G, Senesse P, Bleuse JP, Gourgou S, Sancho-Garnier H (2019). Short- and long-term impact of adapted physical activity and diet counseling during adjuvant breast cancer therapy: the “APAD1” randomized controlled trial. BMC Cancer.

[35] Cornette T, Vincent F, Mandigout S, Antonini MT, Leobon S, Labrunie A (2016). Effects of home-based exercise training on VO_2_ in breast cancer patients under adjuvant or neoadjuvant chemotherapy (SAPA): a randomized controlled trial. Eur J Phys Rehabil Med.

[36] Daley AJ, Crank H, Saxton JM, Mutrie N, Coleman R, Roalfe A (2007). Randomized trial of exercise therapy in women treated for breast cancer. J Clin Oncol.

[37] Foucaut AM, Morelle M, Kempf-Lépine AS, Baudinet C, Meyrand R, Guillemaut S (2019). Feasibility of an exercise and nutritional intervention for weight management during adjuvant treatment for localized breast cancer: the PASAPAS randomized controlled trial. Support Care Cancer.

[38] Husebø AM, Dyrstad SM, Mjaaland I, Søreide JA, Bru E (2014). Effects of scheduled exercise on cancer-related fatigue in women with early breast cancer. ScientificWorldJournal.

[39] Ibrahim M, Muanza T, Smirnow N, Sateren W, Fournier B, Kavan P (2018). The long-term effects of post-treatment exercise on pain in young women with breast cancer. J Commun Support Oncol.

[40] Leach HJ, Potter KB, Hidde MC (2019). A group dynamics-based exercise intervention to improve physical activity maintenance in breast cancer survivors. J Phys Act Health.

[41] May AM, Korstjens I, van Weert E, van den Borne B, Hoekstra-Weebers JE, van der Schans CP (2009). Long-term effects on cancer survivors’ quality of life of physical training versus physical training combined with cognitive-behavioral therapy: results from a randomized trial. Support Care Cancer.

[42] Mazzoni A-S, Brooke HL, Berntsen S, Nordin K, Demmelmaier I (2021). Effect of self-regulatory behaviour change techniques and predictors of physical activity maintenance in cancer survivors: a 12-month follow-up of the Phys-Can RCT. BMC Cancer.

[43] McNeil J, Brenner DR, Stone CR, O’Reilly R, Ruan Y, Vallance JK (2019). Activity tracker to prescribe various exercise intensities in breast cancer survivors. Med Sci Sports Exerc.

[44] Møller T, Andersen C, Lillelund C, Bloomquist K, Christensen KB, Ejlertsen B (2020). Physical deterioration and adaptive recovery in physically inactive breast cancer patients during adjuvant chemotherapy: a randomised controlled trial. Sci Rep.

[45] Mustian KM, Peppone L, Darling TV, Palesh O, Heckler CE, Morrow GR (2009). A 4-week home-based aerobic and resistance exercise program during radiation therapy: a pilot randomized clinical trial. J Support Oncol.

[46] Mutrie N, Campbell A, Barry S, Hefferon K, McConnachie A, Ritchie D (2012). Five-year follow-up of participants in a randomised controlled trial showing benefits from exercise for breast cancer survivors during adjuvant treatment. Are there lasting effects?. J Cancer Surviv..

[47] Penttinen H, Utriainen M, Kellokumpu-Lehtinen P-L, Raitanen J, Sievänen H, Nikander R (2019). Effectiveness of a 12-month exercise intervention on physical activity and quality of life of breast cancer survivors; five-year results of the BREX-study. In Vivo (Athens, Greece).

[48] Pinto BM, Rabin C, Papandonatos GD, Frierson GM, Trunzo JJ, Marcus BH (2008). Maintenance of effects of a home-based physical activity program among breast cancer survivors. Support Care Cancer.

[49] Rogers LQ, Hopkins-Price P, Vicari S, Markwell S, Pamenter R, Courneya KS (2009). Physical activity and health outcomes three months after completing a physical activity behavior change intervention: persistent and delayed effects. Cancer Epidemiol Biomarkers Prev.

[50] Rogers LQ, Courneya KS, Anton PM, Hopkins-Price P, Verhulst S, Vicari SK (2015). Effects of the BEAT Cancer physical activity behavior change intervention on physical activity, aerobic fitness, and quality of life in breast cancer survivors: a multicenter randomized controlled trial. Breast Cancer Res Treat.

[51] Sagen A, Kåresen R, Risberg MA (2009). Physical activity for the affected limb and arm lymphedema after breast cancer surgery A prospective, randomized controlled trial with two years follow-up. Acta Oncol..

[52] Steindorf K, Schmidt ME, Klassen O, Ulrich CM, Oelmann J, Habermann N (2014). Randomized, controlled trial of resistance training in breast cancer patients receiving adjuvant radiotherapy: results on cancer-related fatigue and quality of life. Ann Oncol.

[53] Thorsen L, Dahl AA, Skovlund E, Hornslien K, Fosså SD (2007). Effectiveness after 1 year of a short-term physical activity intervention on cardiorespiratory fitness in cancer patients. J Clin Oncol.

[54] Witlox L, Hiensch AE, Velthuis MJ, Steins Bisschop CN, Los M, Erdkamp FLG (2018). Four-year effects of exercise on fatigue and physical activity in patients with cancer. BMC Med.

[55] Schmidt ME, Wiskemann J, Armbrust P, Schneeweiss A, Ulrich CM, Steindorf K (2015). Effects of resistance exercise on fatigue and quality of life in breast cancer patients undergoing adjuvant chemotherapy: a randomized controlled trial. Int J Cancer.

[56] Vehmanen L, Sievänen H, Kellokumpu-Lehtinen P, Nikander R, Huovinen R, Ruohola J (2021). Five-year follow-up results of aerobic and impact training on bone mineral density in early breast cancer patients. Osteoporos Int.

[57] Kampshoff CS, Jansen F, van Mechelen W, May AM, Brug J, Chinapaw MJM (2014). Determinants of exercise adherence and maintenance among cancer survivors: a systematic review. Int J Behav Nutr Phys Act.

[58] Finne E, Glausch M, Exner AK, Sauzet O, Stölzel F, Seidel N (2018). Behavior change techniques for increasing physical activity in cancer survivors: a systematic review and meta-analysis of randomized controlled trials. Cancer Manag Res.

[59] Howlett N, Trivedi D, Troop NA, Chater AM (2019). Are physical activity interventions for healthy inactive adults effective in promoting behavior change and maintenance, and which behavior change techniques are effective? A systematic review and meta-analysis. Transl Behav Med.

[60] Pinto BM, Dunsiger SI, Kindred MM, Mitchell S (2021) Physical activity adoption and maintenance among breast cancer survivors: a randomized trial of peer mentoring. Ann Behav Med 56(8):842–855 10.1093/abm/kaab078 PMC9345185 34436552

