# Supplementary material for: Long-term effects of exercise interventions on physical activity in breast cancer patients: a systematic review and meta-analysis of randomized controlled trials
Source: Support Care Cancer. 2023 Jan 24;31(2):130. doi: 10.1007/s00520-022-07485-6 (PMC9873715; doi:10.1007/s00520-022-07485-6)
Supplement: Supplementary file 7 — Supplementary file7 (DOCX 21 KB) [file 520_2022_7485_MOESM7_ESM.docx]

**Table S1**: search strategies in the database Pubmed

|  | "Category" | Pubmed | Count at  31.01.2022 |
| --- | --- | --- | --- |
| **1** | **Sustainability** | **maintain*[Tiab] OR maintenance [Tiab] OR sustain*[Tiab] OR upkeep [Tiab] OR uphold [Tiab] OR continue [Tiab] OR long-term [Tiab] OR longterm [Tiab]** | 2,250,199 |
| 2 | Exercise intervention | ((Exercise [mh] OR "exercise"[Tiab] OR physical activity [Tiab] OR sport*[Tiab] OR fitness[tiab])  AND  ((randomized controlled trial[pt] OR controlled clinical trial[pt] OR randomized[tiab] OR randomised[tiab] OR randomly[tiab]) OR (intervention [Tiab] OR training*[Tiab] OR program [Tiab])))  OR  (aerobic exercise*[Tiab] OR endurance exercise*[Tiab] OR resistance exercise*[Tiab] OR strength exercise*[Tiab])  OR  (aerobic training*[Tiab] OR endurance training*[Tiab] OR resistance training*[Tiab] OR strength training*[Tiab]) | 196,057 |
| 3 | Population | "breast"[Tiab] OR "breasts"[Tiab] OR "mamma"[Tiab] OR "mammae"[Tiab] OR "mammas"[Tiab] OR "mammary"[Tiab] | 529,485 |
| 4 | Population | cancer*[tiab] OR neoplas*[ tiab] OR tumor*[ tiab] OR tumour*[ tiab] OR carcinoma*[ tiab] OR malignan*[ tiab] | 3,687,348 |
| 5 | Population | (**Animals [MH] NOT Humans [mh])** | 4,958,947 |
| 6 | Population | (Breast neoplasms[mh] OR ((breast[mh] OR (breast diseases[mh]) AND neoplasms[mh])) AND humans[mh]) | 321,108 |
| 7 | Population | (#3 AND #4 NOT #5) OR #6 | 451,100 |
| 8 | TOTAL | #7 AND #1 AND #2 | 617 |

**Table S2**: search strategies in the database COCHRANE

|  | "Category" | Cochrane - Trials | Count at 31.01.2022 |
| --- | --- | --- | --- |
| **1** | **Sustainability** | **maintain* OR maintenance OR sustain* OR upkeep OR uphold OR continue OR long-term OR longterm** | 336,773 |
| 2 | Exercise intervention | ((Exercise OR "exercise" OR physical activity OR sport* OR fitness)  AND  ((randomized controlled trial OR controlled clinical trial OR randomized OR randomised OR randomly)             OR (intervention OR training* OR program)))  OR  (aerobic exercise* OR endurance exercise* OR resistance exercise* OR strength exercise*)  OR  (aerobic training* OR endurance training* OR resistance training* OR strength training*) | 157,344 |
| 3 | Population | "breast" OR "breasts" OR "mamma" OR "mammae" OR "mammas" OR "mammary" | 53,189 |
| 4 | Population | cancer* OR neoplas* OR tumor* OR tumour* OR carcinoma* OR malignan* | 235,961 |
| 5 | Population | **Animals NOT Humans** | 4,341 |
| 6 | Population | (Breast neoplasms OR ((breast OR (breast diseases) AND neoplasms)) AND humans) | 52,325 |
| 7 | Population | (#3 AND #4 NOT #5) OR #6 | 52,447 |
| 8 | TOTAL | #7 AND #1 AND #2 | 1,177 |

**Table S3**: search strategies in the database Web of Science

|  | "Category" | Web of Science | Count at 31.01.2022 |
| --- | --- | --- | --- |
| **1** | **Sustainability** | **maintain* OR maintenance  OR sustain* OR upkeep OR uphold OR continue OR long-term OR longterm** | 3,572,023 |
| 2 | Exercise intervention | ((Exercise OR "exercise" OR physical activity OR sport* OR fitness)  AND ((randomized controlled trial OR controlled clinical trial OR randomized OR randomised OR randomly)             OR (intervention OR training* OR program)))  OR  (aerobic exercise* OR endurance exercise* OR resistance exercise* OR strength exercise*)  OR  (aerobic training* OR endurance training* OR resistance training* OR strength training*) | 620,197 |
| 3 | Population | "breast" OR "breasts" OR "mamma" OR "mammae" OR "mammas" OR "mammary" | 796,027 |
| 4 | Population | cancer* OR neoplas* OR tumor* OR tumour* OR carcinoma* OR malignan* | 4,744,442 |
| 5 | Population | **Animals NOT Humans** | 903,503 |
| 6 | Population | (Breast neoplasms OR ((breast OR (breast diseases) AND neoplasms)) AND humans) | 172,980 |
| 7 | Population | (3 AND 4 NOT 5) OR 6 | 673,551 |
| 8 | TOTAL | #7 AND #1 AND #2 | 2,165 |

**Table S4:** search strategies in the database EMBASE

|  | "Category" | EMBASE | Count at 13.07.2021 |
| --- | --- | --- | --- |
| **1** | **Sustainability** | **maintain* OR maintenance OR sustain* OR upkeep OR uphold OR continue OR long-term OR longterm** | 3080594 |
| 2 | Exercise intervention | ((Exercise OR "exercise" OR physical activity OR sport* OR fitness)  AND ((randomized controlled trial OR controlled clinical trial OR randomized OR randomised OR randomly)             OR (intervention OR training* OR program)))  OR  (aerobic exercise* OR endurance exercise* OR resistance exercise* OR strength exercise*)  OR  (aerobic training* OR endurance training* OR resistance training* OR strength training*) | 360794 |
| 3 | Population | "breast" OR "breasts" OR "mamma" OR "mammae" OR "mammas" OR "mammary" | 903189 |
| 4 | Population | cancer* OR neoplas* OR tumor* OR tumour* OR carcinoma* OR malignan* | 6511123 |
| 5 | Population | **Animals NOT Humans** | 827052 |
| 6 | Population | (Breast neoplasms OR ((breast OR (breast diseases) AND neoplasms)) AND humans) | 232 |
| 7 | Population | (#3 AND #4 NOT #5) OR #6 | 719751 |
| 8 | TOTAL | #7 AND #1 AND #2 | 1246 |
