# Supplementary material for: Long-term effects of exercise interventions on physical activity in breast cancer patients: a systematic review and meta-analysis of randomized controlled trials
Source: Support Care Cancer. 2023 Jan 24;31(2):130. doi: 10.1007/s00520-022-07485-6 (PMC9873715; doi:10.1007/s00520-022-07485-6)
Supplement: Supplementary file 8 — Supplementary file8 (DOCX 43 KB) [file 520_2022_7485_MOESM8_ESM.docx]

Table S2: Assessment method of the variable physical activity of all studies included in the meta-analyses

| Study | PA outcome(s) | PA measured with | Months after intervention |
| --- | --- | --- | --- |
| Bolam 2019 (Sweden)(33) | MVPA (min/wk) | Accelerometry | 20 |
| Carayol 2019 (France)(57) | MVPA (MET*min/wk) | GPAQ | 6, 12 |
| Cornette 2015 (France)(58) | MVPA (MET*min/wk) | IPAQ | 6.2 |
| McNeil 2019 (Canada)(34) | Total PA (min/day)  MVPA (min/wk) | Accelerometry  Accelerometry | 3 |
| Mutrie 2012 (Scotland)(32) | Total PA (min/week) | SPAQ | 6, 18, 60 |
| Rogers 2009 (United States)(35) | Total PA (Daily PA counts)  MVPA (min/wk) | Accelerometry  Accelerometry | 3 |
| Rogers 2015 (United States)(47) | MVPA (min/wk) | Accelerometry | 3 |
| Schmidt 2017 (Germany)(20) | Total PA (MET*min/wk)  log-transformed | Walking, cycling, exercise  adopted from SQUASH | 3, 12 |
| Steindorf 2014 (Germany)(46) | Total PA (MET*min/wk)  log-transformed | Walking, cycling, exercise  adopted from SQUASH | 2, 6, 12 |
| vanWaart 2015 (Netherlands)(10) | Total PA (min/week) | PASE | 6 |
| Witlox 2018 (Netherlands)(48) | Total PA (min/week) | SQUASH | 4.5, 43.5 |

GPAQ - global physical activity questionnaire, IPAQ - International physical activity questionnaire MET – metabolic equivalent of task, MVPA – moderate-to-vigorous physical activity, PA – Physical activity, PASE - Physical Activity Scale for the elderly, SPAQ – Scottish Physical activity questionnaire, SQUASH- Short Questionnaire to Assess Health-enhancing physical activity
